# Association of *IL-1B+3954* and *IL-1RN* Polymorphisms in Chronic Gastritis and Peptic Ulcer

**Published:** 2018-09

**Authors:** Neda MOTAMEDI RAD, Meysam REZAEISHAHMIRZADI, Sepideh SHAKERI, Mohamad Reza ABBASZADEGAN, Mohammad SHEKARI

**Affiliations:** 1. Dept. of Medical Genetics, School of Medicine, Hormozgan University of Medical Sciences, Bandar-Abbas, Iran; 2. Cancer Molecular Pathology Research Center, Mashhad University of Medical Sciences, Mashhad, Iran; 3. Dept. of Medical Genetics, School of Medicine, Mashhad University of Medical Sciences, Mashhad, Iran; 4. Center for Research of Molecular Medicine, Hormozgan University of Medical Sciences, Bandar-Abbas, Iran

**Keywords:** Interleukin, Gastritis, Peptic ulcer, Polymorphism

## Abstract

**Background:**

*Helicobacter pylori* are the main cause of chronic inflammation and peptic ulcer. We aimed to determine if *IL-1B+*3954 and *IL-1RN* polymorphisms are associated with the risk of chronic gastritis and peptic ulcer in Iranian population.

**Methods:**

In this case-control study, from 198 individuals enrolled by Mohammadi Hospital, Bandar Abbas, southern Iran from 2012 to 2014 and who showed the symptoms of chronic gastritis and 84 with peptic ulcer participated in the case group, two biopsies were taken from the body, antrum, or ulcer edge of each patient. Individuals without chronic gastritis or peptic ulcer were selected as the control group and we also confirmed the presence of anti-*H. pylori* serum IgG in 321 control subjects. *IL-1B+3954C/T* polymorphism was analyzed through PCR-RFLP, while the *IL-1RN* polymorphism was analyzed via PCR-based VNTR.

**Results:**

*IL-1B+3954 TT* was associated with a high risk of gastritis and peptic ulcer [Odds Ratio (OR)]=2.63, 95% Confidence Interval (CI)= (1.47–4.70) (OR=3.40, CI=1.72–6.71) respectively and the *IL-1B*+3954 *T* allele was associated with chronic gastritis (OR=1.64, 95% CI=1.13–2.36). Moreover, patient carrying *IL-1RN L/2* and allele 2 showed an increased risk of peptic ulcer (OR=2.97, CI=1.72–5.11, OR=1.64, CI=1.13–2.36), respectively.

**Conclusion:**

*IL-1B* and *IL-1RN*are associated with an increased risk for chronic gastritis and peptic ulcer disease.

## Introduction

*Helicobacter pylori* is a Gram-negative microaerophilic bacterium, whose infection is one of the major causes of chronic gastritis and peptic ulcer disease (PUD) in human beings (1, 2). A majority of the world population is reported to be infected with *H. pylori*, but only 20% of the infected individuals develop clinically significant disease (3).

While most infected individuals remain asymptomatic, persistent colonization and chronic inflammation increase the risk of developing atrophic gastritis, peptic ulcers, and gastric cancer (4, 5). The clinical outcome of *H. pylori* infection can be determined by the genetic characteristics of the host and the bacteria, and also by environmental factors (6). Recently, inflammatory response is one essential part of the pathogenesis of PUD. Evidence also suggests that inflammatory cytokines, besides the genetic variation in the genes that encode the inflammatory mediators, might play a key role in the development of PUD (7, 8).

The interleukin-1 (*IL-1*) gene cluster on chromo-some 2q contains three related genes within a 430-kb region: *IL-1A*, *IL-1B*, and *IL-1RN*, which encode the pro-inflammation cytokines IL-1a and *IL-1b* and the endogenous receptor antagonist IL-1ra, respectively (9). *IL-1b* is up-regulated in the presence of *H. pylori* and is a potent inhibitor of gastric acid secretion (10). Three di-allelic polymorphisms in *IL-1B* have been reported, all representing C–T base transitions, at positions -511, -31, and +3954 bp. *IL-1RN* has a Penta-allelic 86-bp tandem repeat polymorphism in intron-2, while *IL-1RN* allele 2 has been shown to be associated with enhanced *IL-1b* production (11). Polymorphisms have been reported to be related to variations in the production levels of *IL-1b* and *IL-1ra* (12).

In this study, we investigated whether *IL-1B* (rs1143634) and *IL-1RN* polymorphisms are associated with chronic gastritis or peptic ulcer in Iran.

## Methods

We conducted a case-control study, 282 patients (including 198 patients with chronic gastritis and 84 patients with peptic ulcer) who underwent an endoscopic study in a specialized unit for Gastroenterology Endoscopy in the city of Bandar Abbas in 2012 to 2014, southern Iran were included in this study. All the study participants belonged to Hormozgan Province in Iran, and all of them suffered from functional dyspepsia and epigastric pain. These patients did not receive *H. pylori* eradication therapy. Moreover, we selected those individuals who had no signs of dyspepsia, no history of gastric diseases, and no indication for endoscopy, and used IgG-Anti *H. pylori* ELISA test to diagnose *H. pylori* infection in them. Finally, 321 people were positive for serum *H. pylori* IgG indicating their previous exposure to *H. pylori*. However, they had no signs of gastric diseases so they were selected as the control group.

In both groups, the researcher excluded the subjects under non-steroidal anti-inflammatory drug (NSAID) treatment and those with autoimmune gastritis.

Informed consent was obtained from the participants, and they were informed that the results would be used for developing better treatment strategies for such diseases. Hormozgan Bioethics Committee of the Medical University approved the validity of this study.

Details of the study participants such as diet, socio-demographic factors, family history of gastritis or ulcers, and smoking habits were recorded in surveys.

### Endoscopy and Gastric Histology

Every patient underwent an endoscopy using a video processor and a video gastroscope (Fujinon, Wayne, NJ, USA). In each patient, we took two biopsies from the antrum, corpus, or ulcer edge; one specimen was immediately fixed in formalin for histological testing and the other was placed in distilled water for the diagnostic test. The biopsies collected for the diagnostic test were stored at −20°C until the processing procedures. Furthermore, the histological sections were stained with hematoxylin and eosin and evaluated by a pathologist using updated Sydney System criteria (13). Endoscopic observation and histopathological confirmation were applied to determine the patient’s pathology in the study. And we finally selected 282 individuals *Helicobacter pylori* positive as patient group.

### Serology

Blood samples (5 ml) were collected from all the control group subjects. The serum was tested for IgG anti-*H. pylori* by ELISA; International Immuno-Diagnostics, Foster City, CA, USA) following the manufacturer’s instructions. The sensitivity and specificity of this method was 96%. A subject was considered *H. pylori* positive if we detected antibodies in the serum.

### Analysis of IL1B and IL1RN Genes

**Fig. 1:**
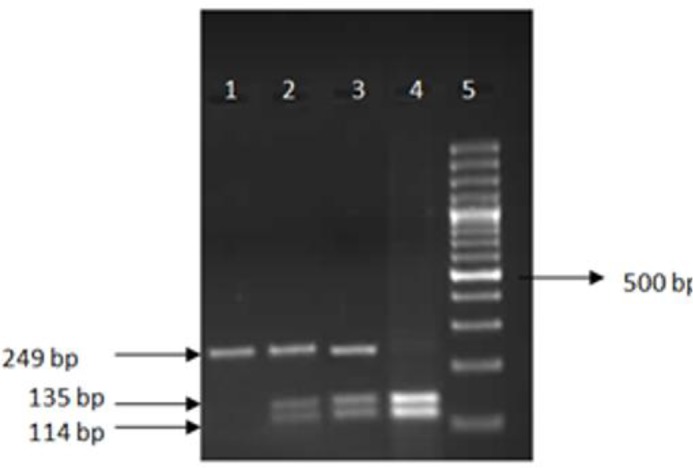
Agarose gel showing interleukin *(IL)-1B TaqI-*restriction patterns in four individuals. Lane 1 homozygous for allele *T*(*TT*) lanes 2,3 show an individual heterozygous (*CT*), and lane 4 homozygous for allele *C*(*CC*), A 100 basepair ladder is shown in the right lane (5)

In this study, genomic DNA was extracted from peripheral blood using the QIA amp DNA Mini Kit. *IL-1B*+3954C/T polymorphisms were analyzed by RFLP with the *TaqI* enzyme (14). The PCR products were analyzed by electrophoresis on a 2% agarose gel stained with 0.1% ethidiumbromide. PCR products were digested with 2 units of *TaqI* for 8 h at 65°C and resulted in an intact fragment of 249 bp (allele T) or in two fragments of 135 and 114 bp (allele C). Fragments were analyzed by electrophoresis on 3% agarose gels stained with 0.1% ethidium bromide (Fig. 1).

### IL-IRN Gene Polymorphism

**Fig. 2:**
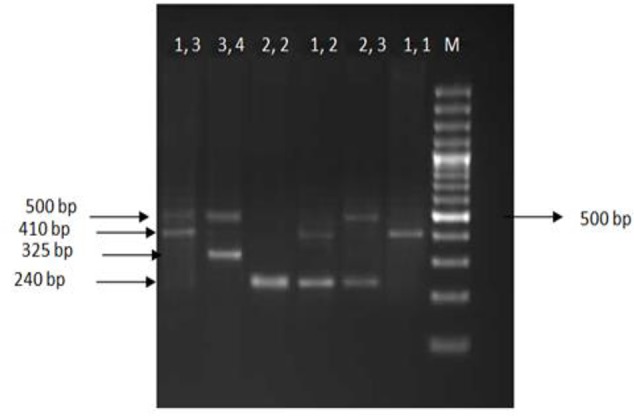
Agarose gel showing six different patterns for interleukin *(IL)-RN* genotypes 1,1. 2,2. 1,2. 1,3. 2,3. And 3,4. A base pair ladder is shown in the right lane (M)

The Penta-allelic variable number of 86-bp VNTR in intron 2 of the *IL-1RN* gene was analyzed by PCR. The oligonucleotides 5′ CTCAGCAACACTCCTAT 3′ and 5′ TCCTGGTCTGCAGGTAA 3′ were used as primers (12). The PCR products resulting in a short allele with two repeats of the 86-bp (IL-1RN*2) or long alleles (IL-1RN*L): allele 1(four repeats), allele 3 (five repeats), allele 4 (three repeats), and allele 5 (six repeats) were analyzed by electrophoresis on 2% agarose gel stained with 0.1% ethidium bromide (Fig. 2).

### Statistical analysis

Hardy–Weinberg equilibrium (HWE) of allelic frequencies at individual loci was assessed by comparing the observed and expected genotype frequencies by incorporating the χ^2^ test (for *IL-1B P*=0.197; for *IL-1RN P*=1.00). For statistical analysis, the χ^2^ test was used to assess the statistical significance of the differences between the genotype and allelic frequencies of the population. Odds ratios (OR) with a 95% confidence interval (CI) were calculated for genotyping objectives. The level of significance was set at *P*<0.05.

## Results

The case group represented 198 individuals with chronic gastritis and 84 patients with peptic ulcer in this study. The average age of the patients with gastritis was 41 yr with an age range of 17 to 85 yr; the peptic ulcer patients had an average age of 42 yr, ranging from 23 to 72 yr. Subjects in the control group had an average age of 41 yr and their age ranged from 21 to 74 yr. There were significant differences among the three groups in family history of ulcer and smoking habits (Table 1).

**Table 1:** Demographic Characteristics of Cases and Controls

	***Control (321) n (%)***	***Gastritis (198) n (%)***	***P***	***Peptic ulcer (84) n (%)***	***P***
Age (yr)	41.8 ±15.9	41.2±16.1	0.96	42.8 ±15.5	0.31
Sex			0.79		0.73
Male	162 (50.4)	103 (52)		40 (47.6)	
Female	159 (49.6)	95 (48)		44(52.4)	
Smoking habit			<0.001		<0.001
Smoker	51 (15.9)	67 (33.8)		34 (40.5)	
Nonsmoker	270 (84.1)	131 (66.2)		50 (59.5)	
Family history ulcer			<0.001		<0.001
Yes	46 (14.3)	66 (33.3)		34 (40.4)	
No	275 (85.7)	123 (66.7)		50 (59.6)	

The relationships between individual *IL-1B* and *IL-1RN* genotypes among patients with a risk of chronic gastritis and peptic ulcer are represented in Tables 2 and 3.

**Table 2:** Genotype and allele frequencies of *IL-1B* and *IL-1RN* polymorphisms in gastritis and control

***Polymorphism***	***Genotype***	***Gastritis (198) n (%)***	***Control (321) n (%)***	***OR^a^ (95%CI)***	***P^a^***
*IL-1B*	*CC*	84 (42.4)	178 (55.5)	1.0	-
	*TC*	79 (39.9)	116 (36.1)	1.40(0.94–2.09)	0.08
	*TT*	35 (17.7)	27 (8.4)	2.63(1.47–4.70)	<0.001
	*TC+TT*	114 (57.6)	143(44.5)	1.64(1.13–2.36)	0.008
*IL-1RN*	L/L	150(75.8)	240(74.8)	1.0	-
	*L/2*	40(20.2)	63 (19.6)	0.99(0.62–1.56)	0.96
	*2/2*	8 (4)	18(5.6)	0.83(0.36–1.89)	0.66
	*L/2+2/2*	48 (24.2)	81(25.2)	0.95(0.62–1.45)	0.83

a Adjusted for smoking and family history ulcer

**Table 3:** Genotype and allele frequencies of *IL-1B* and *IL-1RN* polymorphisms in peptic ulcer and control

***Polymorphism***	***Genotype***	***Peptic ulcer (84) n (%)***	***Control (321) n (%)***	***OR^a^ (95%CI)***	***P^a^***
*IL-1B*	*CC*	42(50)	178(55.5)	1.0	-
	*TC*	19(22.6)	116(36.1)	0.62(0.33–1.16)	0.13
	*TT*	23(27.4)	27(8.4)	3.40(1.72–6.71)	<0.001
	*TC+TT*	42(50)	143(44.5)	0.95(0.62–1.45)	0.83
*IL-1RN*	*L/L*	45(53.6)	240(74.8)	1.0	-
	*L/2*	36(42.9)	63(19.6)	2.97(1.72–5.11)	<0.001
	*2/2*	3(3.5)	18(5.6)	0.87(0.22–3.20)	0.83
	*L/2+2/2*	39(46.4)	81(25.2)	1.64(1.13–2.36)	<0.001

a Adjusted for smoking and Family history ulcer

The genotype frequencies of the *IL-1B* +3954 *TC* and *TT* were significantly different between gastritis and control group (OR=1.40, CI=0.94–2.09, and OR=2.63, CI=1.47–4.70) respectively. Moreover, the allele frequency of *IL-1B* +3954 *T* was significantly different between gastritis and control (OR=1.64, CI=1.13–2.36) but there were no significant differences in any allele or genotype distribution of *IL-1RN* polymorphism between gastritis and control group.

There was significant association between the *IL-1BTT* genotype and peptic ulcer (OR=3.40, CI=1.72–6.71) but there was no significant association between *TC* genotype and *T* allele in peptic ulcer and control group. In peptic ulcer group there was significant association between *L/2* genotype and *2* alleles as compared to control group (OR=2.97, CI=1.72–5.11 and OR=1.64, CI=1.13–2.36). However, there were no differences in the frequency of the *IL-1RN2/2* genotype between peptic ulcer and control.

## Discussion

In this study, homozygous variant (*TT*) of *IL-1B*+3954 gene is associated with peptic ulcer and caring *T* allele (*CT+TT*) is correlated with gastritis. Heterozygous genotype (*L/2*) and caring 2 alleles of IL-1RN (*L/2*+ *2/2*) an increased risk of peptic ulcer.

Different pathological mechanisms related to the progression of chronic gastritis and peptic ulcer is suggested (12). *H. pylori* infection can cause hypochlorhydria, gastric atrophy, and eventually peptic ulcer and cancer. In fact, the interaction between the microorganism and the host immune system will lead to diseases. After *H. pylori* infection, the local immune cells are activated and exhibit immune responses affecting the disease process. For example, increased production of pro-inflammatory cytokines in the mucus (such as *IL-1*, *IL-6*, *IL-10*, *TNF-α*, and interferon-γ) of those who are at risk of *H. pylori* infection is associated with gastritis induced by *H. pylori* and the severity of inflammation (15). *IL-1B* is a member of the *IL-1* family and a pro-inflammatory cytokine involved in the immune defense against infection. *IL-1B* expression increases the expression of factors adhered to vascular endothelial cells that cause the leukocytes to extravagate to the site of infection and resets the temperature regulation center in the hypothalamus which leads to increased body temperature. These are the activities of body immune system against infection. In addition, for the role of *IL-1B* in adjusting a secretion of acid and protecting cell activities in gastric mucus, there is a body of research related to *IL-1B* polymorphism, peptic and gastric ulcer. Also, it is reported that *IL-1B* is a cell-protecting molecule and strongly limits the gastric acid secretion (16). Therefore, *IL-1* gene polymorphisms may be associated with the severity of inflammatory diseases, autoimmune diseases, such as rheumatoid arthritis, and cancers, including gastric cancer (17).

In the context of such subjects, the effects of polymorphism in two cytokine genes, the pro-inflammatory cytokine *IL-1B* and anti-inflammatory cytokine *IL-1RN*, in relation to the development of peptic ulcer and chronic gastritis were analyzed. In this study, a significant association was observed between *IL-1B+3954TT* and *T* allele genotype and patients with gastritis and *TT* genotype and peptic ulcer compared with the control group. Since the presence of *TT* polymorphism in *IL-1B* has been reported as associated with increased gene expression (18) and this increased expression enhances the severity of the gastric mucosal inflammatory responses (19), carriers of the *TT* genotype are susceptible to peptic ulcer and gastritis. Similar results have been obtained from the populations of Poland(20), African Americans and Caucasian (21), but this association was not found in the Chinese population (22). This difference in results can be due to different genetic backgrounds in different ethnic groups.

In addition, we conducted on *IL-1RN* polymorphism, a significant association was also observed between the genotype *L/2* and carriers of the allele *2* and peptic ulcer. *IL-1RN* is involved in gastric diseases. The allele *2* is associated with increased risk of precancerous lesions indicating the role of this polymorphism in the early stages of gastric carcinogenesis (23). In another study on the Caucasian population, the allele *2* had a significant association with increased risk of gastric cancer (24), but this association was not observed in the Korean population (11). In addition, in a study on the cytokine polymorphisms in which a population of Southern Brazil was involved, a significant association was observed between the frequency of the allele *2* and development of gastric cancer (25). These differences may be due to lower frequency of the risk allele in some populations, such as the Asian population.

In this study, the frequencies of allele 2 and *L/2* were 46.4%, 42.9% and 25.2%, 19.6% in patients with peptic ulcer and control individuals, respectively. Since the allele 2 is associated with increased production of the *IL-1B*, which limits the amount of gastric acid and that acid reduction is favorable for bacterial colonization, the allele *2* can play a role in the development of gastric diseases with the presence of *H. pylori* (19).

## Conclusion

This study supports the previous evidence on the role of inflammatory factors in development of gastric diseases in the Caucasian population which can potentially help to identify the patients at risk and to apply new methods of treatment in the future.

## Ethical considerations

Ethical issues (Including plagiarism, informed consent, misconduct, data fabrication and/or falsification, double publication and/or submission, redundancy, etc.) have been completely observed by the authors.

## References

[1] Barbosa HPM Martins LC dos Santos SEB (2009). Interleukin-1 and TNF-α polymorphisms and *Helicobacter pylori* in a Brazilian Amazon population. World J Gastroenterol, 15 (12): 1465–71. 19322919 10.3748/wjg.15.1465 PMC2665140

[2] Qadri Q Rasool R Afroze D (2014). Study of *TLR4* and *IL-8* Gene Polymorphisms in *H. pylori*-Induced Inflammation in Gastric Cancer in an Ethnic Kashmiri Population. Immunol Invest, 43 (4): 324–36. 24295404 10.3109/08820139.2013.854378

[3] Chen A Li CN Hsu PI (2004). Risks of interleukin-1 genetic polymorphisms and *Helicobacter pylori* infection in the development of gastric cancer. Aliment Pharmacol Ther, 20 (2): 203–11. 15233701 10.1111/j.1365-2036.2004.01826.x

[4] Rad R Prinz C Neu B (2003). Synergistic effect of *Helicobacter pylori* virulence factors and interleukin-1 polymorphisms for the development of severe histological changes in the gastric mucosa. J Infect Dis, 188 (2): 272–81. 12854083 10.1086/376458

[5] Wang YM Li ZX Tang FB (2016). Association of genetic polymorphisms of interleukins with gastric cancer and precancerous gastric lesions in a high-risk Chinese population. Tumour Biol, 37 (2): 2233–42. 26358252 10.1007/s13277-015-4022-x

[6] Peek RM Blaser MJ (2002). *Helicobacter pylori* and gastrointestinal tract adenocarcinomas. Nat Rev Cancer, 2 (1): 28–37. 11902583 10.1038/nrc703

[7] Yin YW Hu AM Sun QQ (2013). Association between interleukin-8 gene-251 *T/A* polymorphism and the risk of peptic ulcer disease: A meta-analysis. Hum Immunol, 74 (1): 125–30. 23000201 10.1016/j.humimm.2012.09.006

[8] Kumar S Kumari N Mittal RD (2015). Association between pro-(*IL-8*) and anti-inflammatory (*IL-10*) cytokine variants and their serum levels and *H. pylori*-related gastric carcinogenesis in northern India. Meta Gene, 6: 9–16. 26380815 10.1016/j.mgene.2015.07.008 PMC4556814

[9] Dinarello CA (1996). Biologic basis for interleukin-1 in disease. Blood, 87 (6): 2095–147. 8630372

[10] Ramis IB Vianna JS Halicki PCB (2015). Relationship of interleukin-1B gene promoter region polymorphism with *Helicobacter pylori* infection and gastritis. J Infect Dev Ctries, 9 (10): 1108–16. 26517486 10.3855/jidc.6123

[11] Lee SG Kim B Choi W (2003). Lack of association between pro-inflammatory genotypes of the interleukin-1 (*IL-1B-31 C/+* and *IL-1RN* 2/* 2*) and gastric cancer/duodenal ulcer in Korean population. Cytokine, 21 (4): 167–71. 12788304 10.1016/s1043-4666(03)00032-2

[12] GarciaGonzalez M Lanas A Santolaria S (2001). The polymorphic *IL-1B* and *IL-1RN* genes in the aetiopathogenesis of peptic ulcer. Clin Exp Immunol, 125 (3): 368–75. 11531943 10.1046/j.1365-2249.2001.01593.x PMC1906147

[13] Manfred Stolte M (2001). The updated Sydney system: classification and grading of gastritis as the basis of diagnosis and treatment. Can J Gastroenterol, 15 (9): 591–8. 11573102 10.1155/2001/367832

[14] Pociot F Mølvig J Wogensen L (1992). A *Taql* polymorphism in the human interleukin-1β (*IL-1β*) gene correlates with *IL-1β* secretion in vitro. Eur J Clin Invest, 22 (6): 396–402. 1353022 10.1111/j.1365-2362.1992.tb01480.x

[15] He B Pan Y Xu Y (2012). Increased Risk for Gastric Cancer in Carriers of the Lymphotoxin-α+ 252G Variant Infected by *Helicobacter pylori*. Genet Test Mol Biomarkers, 16 (1): 9–14. 21793721 10.1089/gtmb.2011.0078

[16] Zhang BB Wang J Bian DL Chen X-Y (2012). No association between *IL-1β-31 C/T* polymorphism and the risk of duodenal ulcer: a meta-analysis of 3,793 subjects. Hum Immunol, 73 (11): 1200–6. 22917539 10.1016/j.humimm.2012.08.006

[17] He BS Pan YQ Xu YF (2011). Polymorphisms in interleukin-1B (*IL-1B*) and interleukin 1 receptor antagonist (*IL-1RN*) genes associate with gastric cancer risk in the Chinese population. Dig Dis Sci, 56 (7): 2017–23. 21243433 10.1007/s10620-010-1557-y

[18] Resende RG Guimarães Abreu MHN de Souza LN (2013). Association between *IL1B* (+ 3954) polymorphisms and *IL-1β* levels in blood and saliva, together with acute graft-versus-host disease. J Interferon Cytokine Res, 33 (7): 392–7. 23659674 10.1089/jir.2012.0111

[19] ElOmar EM Carrington M Chow WH (2000). Interleukin-1 polymorphisms associated with increased risk of gastric cancer. Nature, 404 (6776): 398–402. 10746728 10.1038/35006081

[20] Hnatyszyn A Wielgus K Kaczmarek Rys M (2013). Interleukin-1 gene polymorphisms in chronic gastritis patients infected with *Helicobacter pylori* as risk factors of gastric cancer development. Arch Immunol Ther Exp (Warsz), 61 (6): 503–12. 23995914 10.1007/s00005-013-0245-y PMC3898137

[21] Zabaleta J Camargo MC Piazuelo MB (2006). Association of interleukin-1β gene polymorphisms with precancerous gastric lesions in African Americans and Caucasians. Am J Gastroenterol, 101 (1): 163–71. 16405550 10.1111/j.1572-0241.2006.00387.x

[22] Zeng ZR Hu PJ Hu S (2003). Association of interleukin 1B gene polymorphism and gastric cancers in high and low prevalence regions in China. Gut, 52 (12): 1684–9. 14633943 10.1136/gut.52.12.1684 PMC1773879

[23] Peleteiro B Lunet N Carrilho C (2010). Association between cytokine gene polymorphisms and gastric precancerous lesions: systematic review and meta-analysis. Cancer Epidemiol Biomarkers Prev, 19 (3): 762–76. 20200422 10.1158/1055-9965.EPI-09-0917

[24] Xue H Lin B Ni P (2010). Interleukin-1B and interleukin-1 RN polymorphisms and gastric carcinoma risk: A meta-analysis. J Gastroenterol Hepatol, 25 (10): 1604–17. 20880168 10.1111/j.1440-1746.2010.06428.x

[25] Rocha GA Guerra JB Rocha AMC (2005). *IL1RN* polymorphic gene and cagA-positive status independently increase the risk of noncardia gastric carcinoma. Int J Cancer, 115 (5): 678–83. 15704154 10.1002/ijc.20935

